# Comparative evaluation of mesenchymal stromal cells from umbilical cord and amniotic membrane in xeno-free conditions

**DOI:** 10.1186/s12860-018-0178-8

**Published:** 2018-12-13

**Authors:** Yongxu Mu, Xiaoyun Wu, Zhiming Hao

**Affiliations:** 1 Department of Rheumatology, the First Affiliated Hospital of Xi’an Jiaotong University, Xi’an, Shaanxi Pvovince China; 2 Department of Interventional Treatment, the First Affiliated Hospital of Baotou Medical College, Inner Mongolia University of Science and Technology, Baotou, Inner Mongolia China; 3 Department of Technology, Stem Cell Medicine Engineering & Technology Research Center of Inner Mongolia, Huhhot, Inner Mongolia China; 4 Department of Research and Development, Beijing Jingmeng Stem Cell Technology. Co. Ltd., Beijing, China

**Keywords:** Mesenchymal stromal cells, Amnion, Umbilical cord, Platelet lysate, Characteristics, Immunomodulatory

## Abstract

**Background:**

Within the past years, umbilical cord (UC) and amniotic membrane (AM) expanded in human platelet lysate (PL) have been found to become increasingly candidate of mesenchymal stromal cells (MSCs) in preclinical and clinical studies. Different sources of MSCs have different properties, and lead to different therapeutic applications. However, the similarity and differences between the AMMSCs and UCMSCs in PL remain unclear.

**Results:**

In this study, we conduct a direct head-to-head comparison with regard to biological characteristics (morphology, immunophenotype, self-renewal capacity, and trilineage differentiation potential) and immunosuppression effects of AMMSCs and UCMSCs expanded in PL. Our results indicated that AMMSCs showed similar morphology, immunophenotype, proliferative capacity and colony efficiency with UCMSCs. Moreover, no significantly differences in osteogenic, chondrogenic and adipogenic differentiation potential were observed between the two types of cells. However, AMMSCs exhibited higher PGE_2_ expression and IDO activity compared with UCMSCs when primed by IFN-γ and (or) TNF-α induction, and AMMSCs showed a higher inhibitory effect on PBMCs proliferation than UCMSCs.

**Conclusion:**

The results suggest that AMMSCs expanded in PL showed similar morphology, immunophenotype, self-renewal capacity, and trilineage differentiation potential with UCMSCs. However, AMMSCs possessed superior immunosuppression effects in comparison with UCMSCs. These results suggest that AMMSCs in PL might be more suitable than UCMSCs for treatment of immune diseases. This work provides a novel insight into choosing the appropriate source of MSCs for treatment of immune diseases.

**Electronic supplementary material:**

The online version of this article (10.1186/s12860-018-0178-8) contains supplementary material, which is available to authorized users.

## Background

Mesenchymal stromal cells (MSCs) are popular cells for regenerative medicine due to their capacity of extensive self-renewal, multilineage differentiation potential, and immunosuppressive effects [1]. Due to their low proportion in human tissues, extensive in vitro expansion is necessary to attain sufficient cell numbers for MSCs-based therapies. Traditionally, culture media for the isolation and expansion of MSCs in basic research and most clinical studies usually comprise fetal bovine serum (FBS). FBS is an animal-derived product, and its usage has raised safety concern [2]. The current regulatory settings aiming to minimize the usage of FBS have reinforced an intensive search for possible substitutes [2]. Over the last decade, many laboratories adapt their “xenogen-free or animal-free” culture condition to human platelet lysate (PL), which allows expansion and clinical grade production of MSCs for clinical applications [3]. The usage of PL in MSCs culture could provide advantages as follows: (1) PL as a human reagent, is the absence of any risk of xenogeneic immune reactions or transmission of bovine pathogens [4]. (2) MSCs in PL-supplemented medium display a smaller in size and more elongated morphology, faster attachment and migration rate, higher proliferation effect, and greater osteogenic and chondrogenic differentiation potential [4]. Therefore, PL has been widely used as a FBS substitute for clinical-scale expansion of MSCs from various sources, although the biological characteristics and (or) therapeutic potential can be changed in PL [5].

MSCs are first identified and isolated from bone marrow, which has emerged as the most common source in MSCs-based therapies and tissue engineering [6]. In recent years, umbilical cord (UC) and amniotic membrane (AM) appear to be more promising sources of MSCs [7]. Both UC and AM are of foetal origin from perinatal tissues, and provide more primitive cells, which exhibit superior cell activity including higher self-renewal capacity, greater differentiation potential and lower immunogenicity when compared with bone marrow [8–10]. Another important advantage of perinatal tissues is that they are usually discarded as a medical waste, can be obtained easily without ethical constraints [8]. Accumulating evidences have shown that UC derived MSCs (UCMSCs) may have a therapeutic advantage for MSCs-based therapies because of their primitive features [11, 12]. Within the past years, AM has been also found to become increasingly candidate of MSCs in preclinical and clinical studies [7].

Besides culture condition, source-dependent differences in biological characteristics of MSCs have recently emerged and lead to different therapeutic applications [13]. The biological characteristics have been compared between UCMSCs and AM derived MSCs (AMMSCs) in FBS-supplemented medium, but the results are in conflict [14–17]. The similarity and differences between both types of cells in PL are not clear to date. Moreover, optimal sources for treatment of immune diseases remain to be identified. In this study we conduct a direct head-to-head comparison with regard to their morphology, immunophenotype, self-renewal capacity, trilineage differentiation potential, and immunosuppressive effects.

## Methods

### PL preparation

**Fig. 1:**
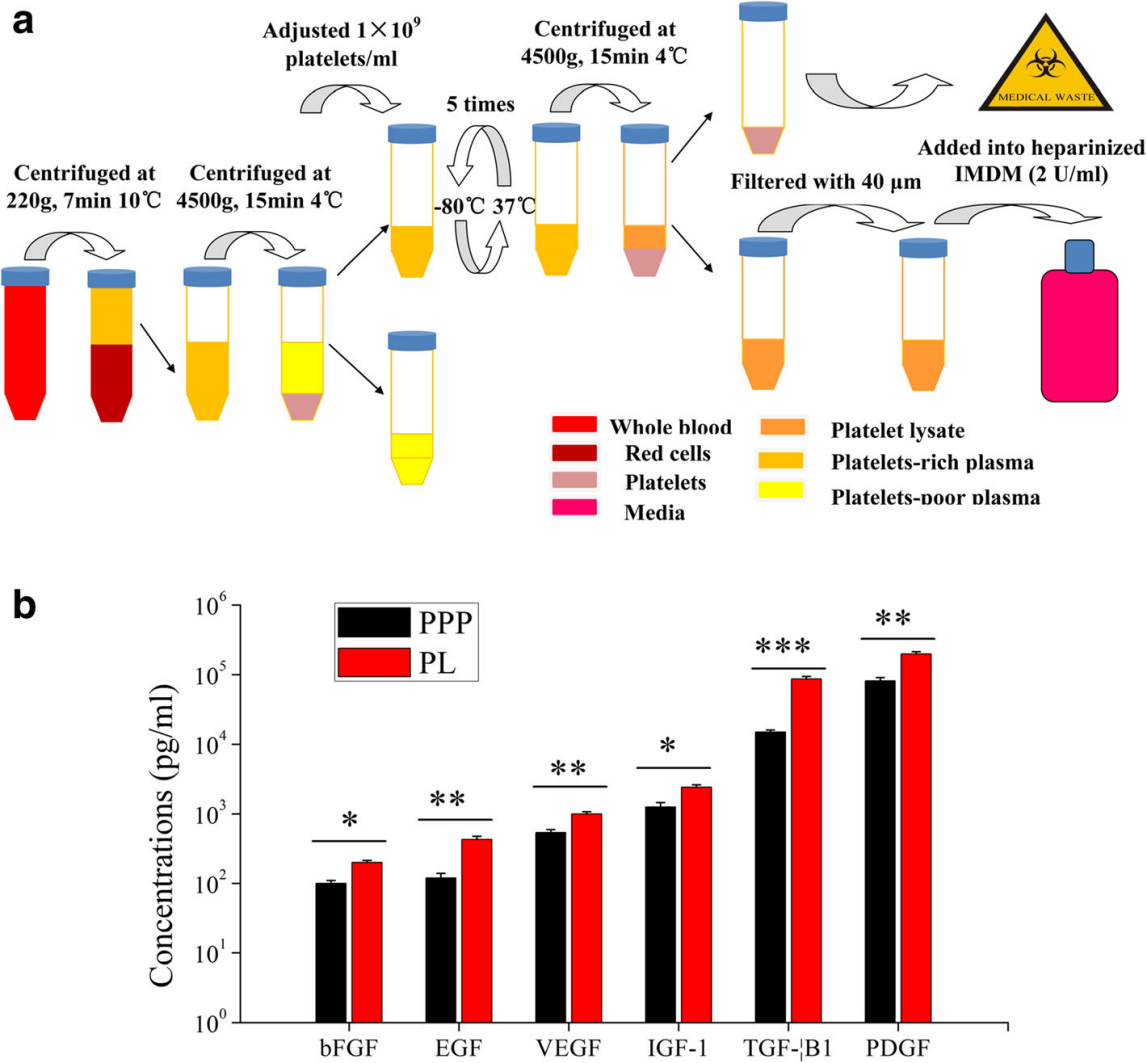
Platelet lysis released sufficient amounts of growth factors in PL. **a** Schematic overview of preparation of PL. PRP was generated from peripheral blood through a series of centrifugations. Based on platelet counts (10^9^ platelets/ml) after the centrifugation step, sufficient PPP was removed to achieve PRP. PL was prepared from PRP by a simple freeze-thawing procedure. **b** The quantification of growth factors in PL and PPP was determined using ELISA. Bars represented means ± SD. *n* = 5; *p < 0.05, **p < 0.01, and ***p < 0.005

PL was prepared from whole blood unit that was harvested from healthy donor between 18 and 65 years old with some modifications as described previously [18]. Briefly, platelet-rich plasma (PRP) was generated by enriching whole blood platelet concentration using a series of centrifugations, and standardized to a concentration of 1 × 10^9^ platelets/ml by removing excess platelet-poor plasma (PPP). PL was prepared from PRP by a simple freeze-thawing procedure, centrifuged to remove the platelet fragments, and filtered further using a 40-μM filter (BD Biosciences, Franklin Lakes, USA). The preparation methodology is illustrated in Fig. 1a. At least 10 thawed PL were pooled to prepare a standardized pooled PL, and added to heparinized iscove’s modified dulbecco’s medium (IMDM, 2 U/ml).

### Quantification of growth factors in PL and PPP

Several growth factors including platelet-derived growth factor (PDGF), basic fibroblast growth factor (bFGF), epithelial growth factor (EGF), transforming growth factor-beta 1 (TGF-β1), insulin-like growth factor-1 (IGF-1) and vascular endothelial growth factor (VEGF) have already been known to be required for MSCs growth in vitro, so the quantification of these growth factors in PL and PPP was determined using enzyme linked immunosorbent assay (ELISA) according to the manufacturer’s instruction (Sino Biological Inc., Beijing, China).

### Human UCMSCs and AMMSCs cultures

All human UC (*n* = 5) and AM (*n* = 5) samples were obtained from healthy, full-term, complicated pregnancies with informed consent, and the study was approved by the Ethics Committee of the First Affiliated Hospital of Baotou Medical College. UC was sectioned (5 to 10 cm long), and umbilical arteries and vein were removed. The AM was mechanically peeled off from the placenta. Both UC and AM were washed with phosphate buffered saline (PBS) to remove excessive blood and cellular debris, minced into approximately 1 × 1 mm^3^ pieces. AM was incubated in 0.25% trypsin solution for 60 min in 37 °Cto eliminate epithelial cells. An enzyme cocktail (hyaluronidase 5 U/ml, collagenase 125 U/ml and dispase 50 U/ml; Sigma, St. Louis, MO, USA) was used to digest the pieces for 60 min with gentle agitation at 37 °C. The total nucleated cells were plated at a concentration of 2 × 10^5^/ cm^2^ in 5% PL-supplemented media. The fresh medium was changed twice per week. When reaching 80% confluence, cells were replated at 2000 cells/cm^2^.

### Colony-forming unit fibroblast (CFU-F) assays

Total nucleated cells were plated in six-well culture plates in 5% PL-supplemented media at densities of 1 × 10^5^ per well. On day 14, the cell layer was fixed with methanol and stained with crystal violet. Individual colonies composed of at least 50 cells were counted.

### Proliferation studies

The population doubling (PD) was determined using the following formula:

$$ PD=\frac{\left[\log 10(Nh)-\log 10(Np)\right]}{\log 10(2)} $$

*N*_*h*_ is the harvested cells number and *N*_*p*_ is the initial cells number. The PD was calculated and added to the PD of previous passages to generate cumulative population doublings (CPD) of each passage.

### Flow cytometry analysis

Standard flow cytometry analysis was performed to determine the defined MSCs markers for UCMSCs and AMMSCs expanded in PL-supplemented media at passage 5 by using Human MSC Analysis Kit (BD Biosciences, Franklin Lakes, NJ, USA). Data were acquired and analyzed using a FACScan flow cytometer running CellQuest software.

### Multilineage differentiation and staining assay

Osteogenic, chondrogenic and adipogenic differentiation capacity of UCMSCs and AMMSCs was assessed. Both UCMSCs and AMMSCs expanded in PL-supplemented media at passage 5 were induced toward an osteogenic, chondrogenic or adipogenic lineages by using StemPro Osteogenesis, Adipogenesis or Chondrogenesis Differentiation Kit (Gibco, Grand Island, NY, USA), respectively. Osteogenic, chondrogenic and adipogenic differentiation was detected by alizarin red, alcian blue, and oil red O staining.

### Real-time polymerase chain reaction (PCR) analysis

Real-time PCR was performed as described previously [19]. Primers were used in Additional file 1. Platinum SYBR Green qPCR SuperMix-UDG (Invitrogen Darmstadt, Germany) was used under the following conditions: 50°Cfor 2 min, 95°Cfor 2 min, and then 95 °C for 3 s and 60 °C for 30 s for a total of 40 cycles. Result was analyzed using the 2^-∆Ct^ method.

**Table 1:** Surface marker expression levels of UCMSCs and AMMSCs in PL

Surface marker	Expression level (%)		*P* value
	UCMSCs	AMMSCs	
CD73	96.82 ± 2.35	97.48 ± 1.74	*P*>0.05
CD90	99.24 ± 1.53	99.76 ± 1.29	*P*>0.05
CD105	97.44 ± 2.08	97.74 ± 3.28	*P*>0.05
CD14	1.43 ± 0.52	1.67 ± 0.28	*P*>0.05
CD19	0.96 ± 0.24	0.75 ± 0.31	*P*>0.05
CD34	1.71 ± 0.60	1.59 ± 0.53	*P*>0.05
CD45	1.73 ± 0.73	1.41 ± 1.15	*P*>0.05
HLA-DR	1.26 ± 1.07	1.91 ± 1.46	*P*>0.05

Data are expressed as mean ± SD

### Immune function assay

Both UCMSCs and AMMSCs cultured in PL-supplemented media at passage 5 were treated with 15 ng/ml interferon-gamma (IFN-γ) and/or 15 ng/ml tumor necrosis factor-alpha (TNF-α), for 48 h. Prostaglandin E_2_ (PGE_2_) and TGF-β1 concentrations in conditioned medium were quantified using ELISA (Sino Biological Inc., Beijing, China) according to the manufacturer’s instructions.: Indoleamine 2, 3-dioxygenase (IDO) activity was evaluated using kynurenine level.

### Mixed lymphocyte culture assays

Both UCMSCs and AMMSCs at passage 5 were treated with mitomycin C (50 μg/ml for 60 min) to inhibit the proliferation. 1 × 10^4^ UCMSCs or AMMSCs were co-cultured with 4 × 10^4^ human peripheral blood mononuclear cells (PBMCs) in 96-well culture plate with 1 μg/ml anti-CD3, anti-CD28 and interleukin-2 (Gibco, Grand Island, NY, USA) for 48, 72 and 96 h. Cell Counting Kit-8 assay (Dojindo, Japan) was performed according to the manufacturer’s instruction.

### Statistical analysis

Data were expressed as the means ± SD. Statistical analysis was performed with Student’s t-test or One Way ANOVA to compare differences between groups. A *P* value less than 0.05 was considered statistically significant.

## Results

### Platelet lysis released sufficient amounts of growth factors

The results showed that PPP contained a certain concentration of PDGF, bFGF, EGF, TGF-β1, IGF-1 and VEGF, and freeze-thaw rupture of platelets had greatly elevated levels of growth factors in PL (bFGF and IGF-1, both *P* < 0.05; PDGF, EGF, and VEGF, all *P* < 0.01; TGF-β1, *P* < 0.005; Fig. 1b).

### AMMSCs in PL showed similar morphology with UCMSCs

**Fig. 2:**
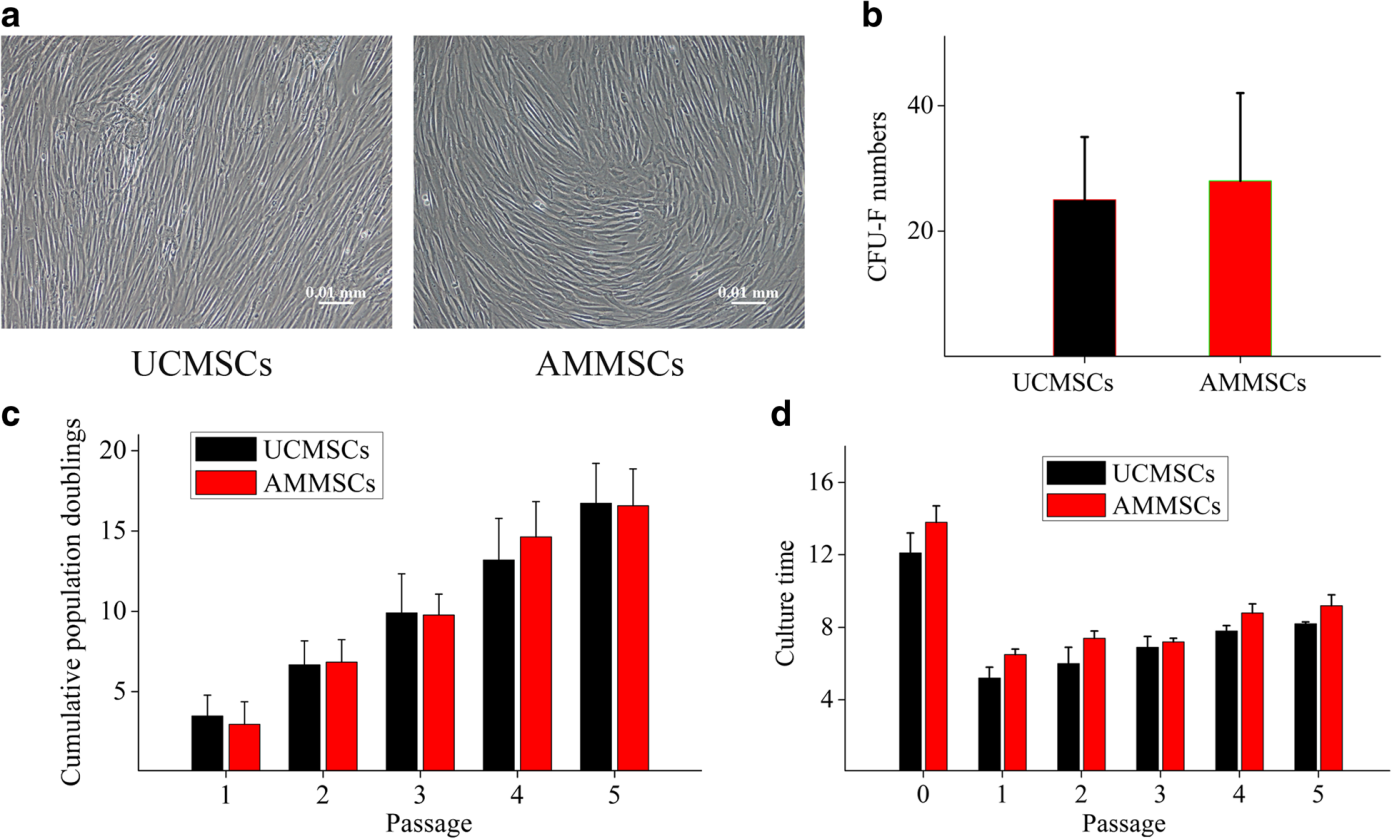
AMMSCs in PL showed similar morphology and self-renewal capacity with UCMSCs. **a** Both UCMSCs and ATMSCs expanded in PL showed fibroblast-like morphologies. Scale bar: 10 μm. **b** Clone-forming ability of UCMSCs and AMMSCs was assessed by CFU-F counts per 1 × 10^5^ TNCs. The proliferation capacity of UCMSCs and AMMSCs was assessed by CPD **c** and culture time **d** of each passage. Bars represented means ± SD; *n* = 5

The UCMSCs and ATMSCs expanded in PL showed fibroblast-like morphologies with parallel or vortex-like patterns, and no morphologic difference was observed between AMMSCs and UCMSCs at passage 5 (Fig. 2a).

### AMMSCs in PL showed similar self-renewal capacity with UCMSCs

CFU-F analysis showed no significant difference in colony counts between AMMSCs and UCMSCs (*P* > 0.05, Fig. 2b). CPD analysis showed that AMMSCs possessed similar CPD numbers (all *P* > 0.05, Fig. 2c) and culture times (all *P* > 0.05, Fig. 2d) for each passage with UCMSCs, indicating that AMMSCs had similar self-renewal capacity with UCMSCs.

### AMMSCs in PL showed similar immunophenotype with UCMSCs

Flow cytometry analysis showed that both UCMSCs and AMMSCs expanded in PL, expressed high levels of CD73, CD90 and CD105 and lacked expression of CD14, CD19, CD34, CD45 and HLA-DR surface markers. Moreover, there were no significant differences between AMMSCs and UCMSCs (all *P* > 0.05, Table 1).

### AMMSCs in PL showed similar trilineage differentiation potential with UCMSCs

**Fig. 3:**
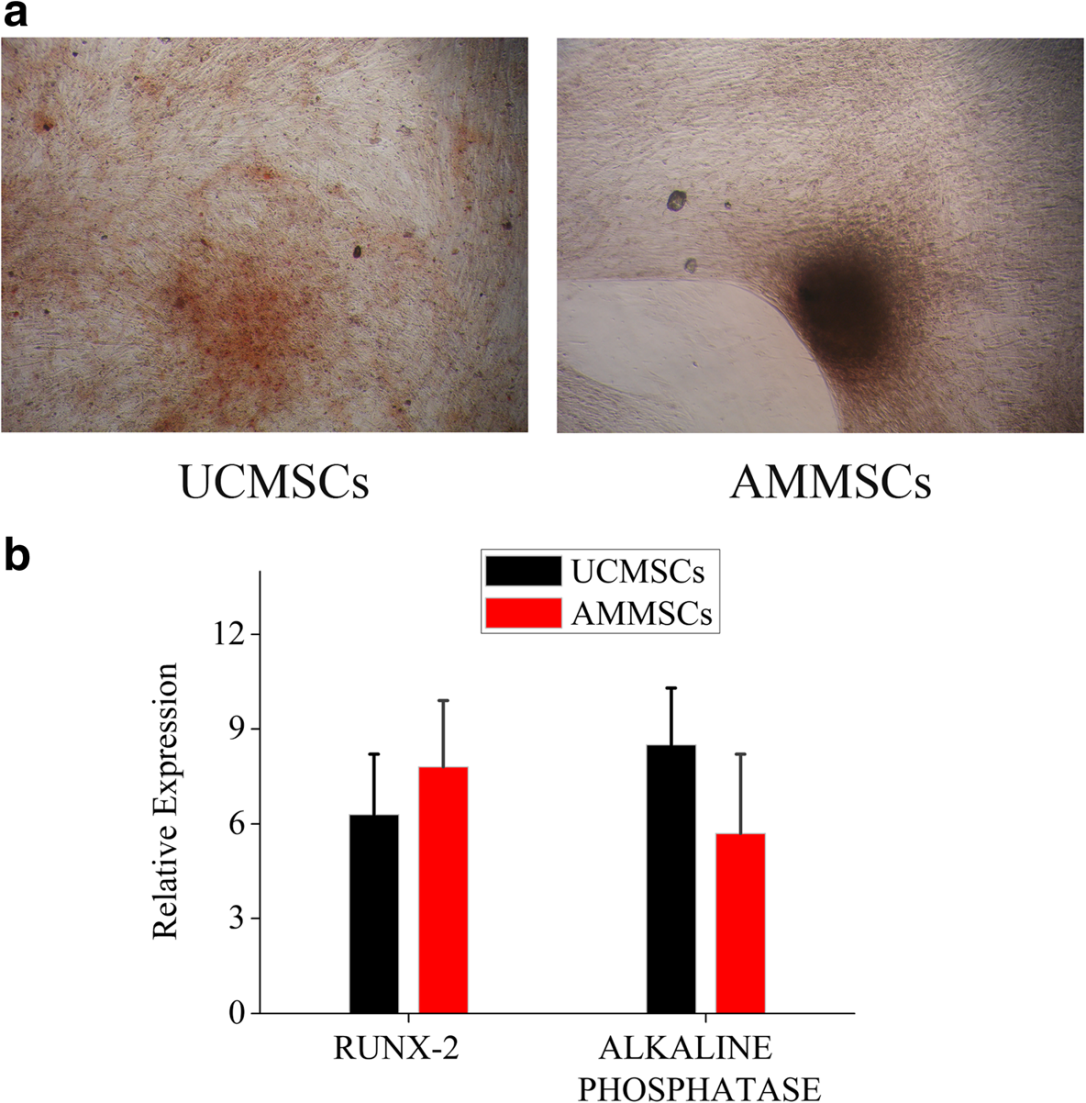
AMMSCs in PL showed similar osteogenic differentiation potential with UCMSCs. **a** The osteogenic differentiation of AMMSCs and UCMSCs was confirmed by Alizarin Red staining of mineralization. Magnification × 100. **b** Comparative investigation of osteogenic differentiation capability of UCMSCs and AMMSCs was assessed by quantitative analysis of RUNX-2 and alkaline phosphatase mRNA expression. Bars represented means ± SD; *n* = 5

The osteogenic differentiation of AMMSCs and UCMSCs was confirmed by alizarin red staining of mineralization (Fig. 3a). The osteogenic AMMSCs exhibited a statistically similar mRNA expression of RUNX-2 and alkaline phosphatase with UCMSCs, indicating that AMMSCs in PL had similar osteogenic differentiation potential with UCMSCs (Both *P* > 0.05, Fig. 3b).

**Fig. 4:**
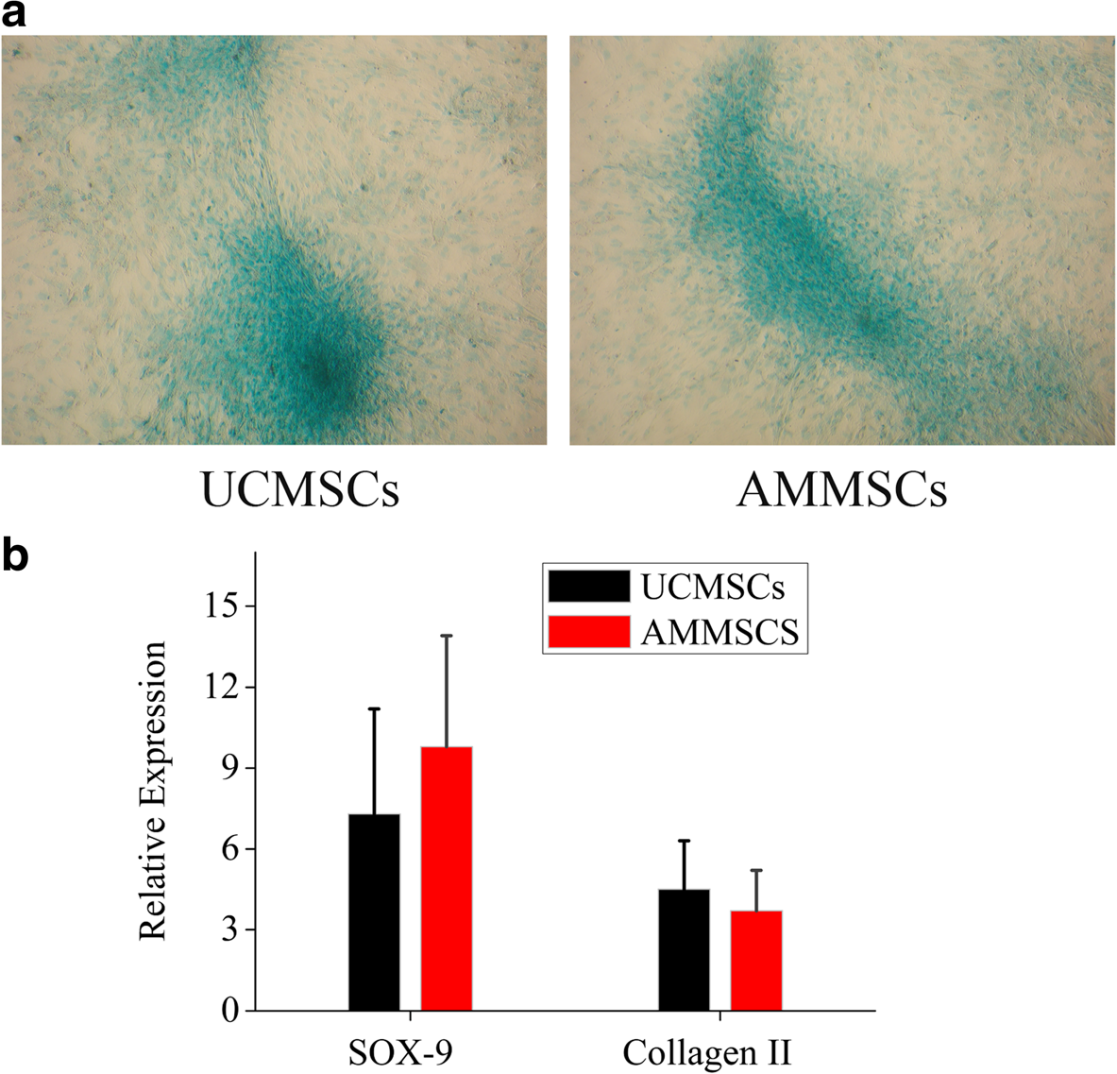
AMMSCs in PL showed similar chondrogenic differentiation potential with UCMSCs. **a** The chondrogenic differentiation of AMMSCs and UCMSCs was confirmed by Alcian Blue staining of glycosaminoglycans. Magnification × 100. **b** Comparative investigation of chondrogenic differentiation capability of UCMSCs and AMMSCs was assessed by quantitative analysis of SOX-9 and collagen II mRNA expression. Bars represented means ± SD; *n* = 5

The chondrogenic differentiation of AMMSCs and UCMSCs was confirmed by alcian blue staining of glycosaminoglycans (Fig. 4a). The chondrogenic AMMSCs exhibited statistically similar mRNA expression of SOX-9 and collagen II with UCMSCs, indicating that AMMSCs in PL had similar chondrogenic differentiation potential with UCMSCs (Both *P* > 0.05, Fig. 4b).

**Fig. 5:**
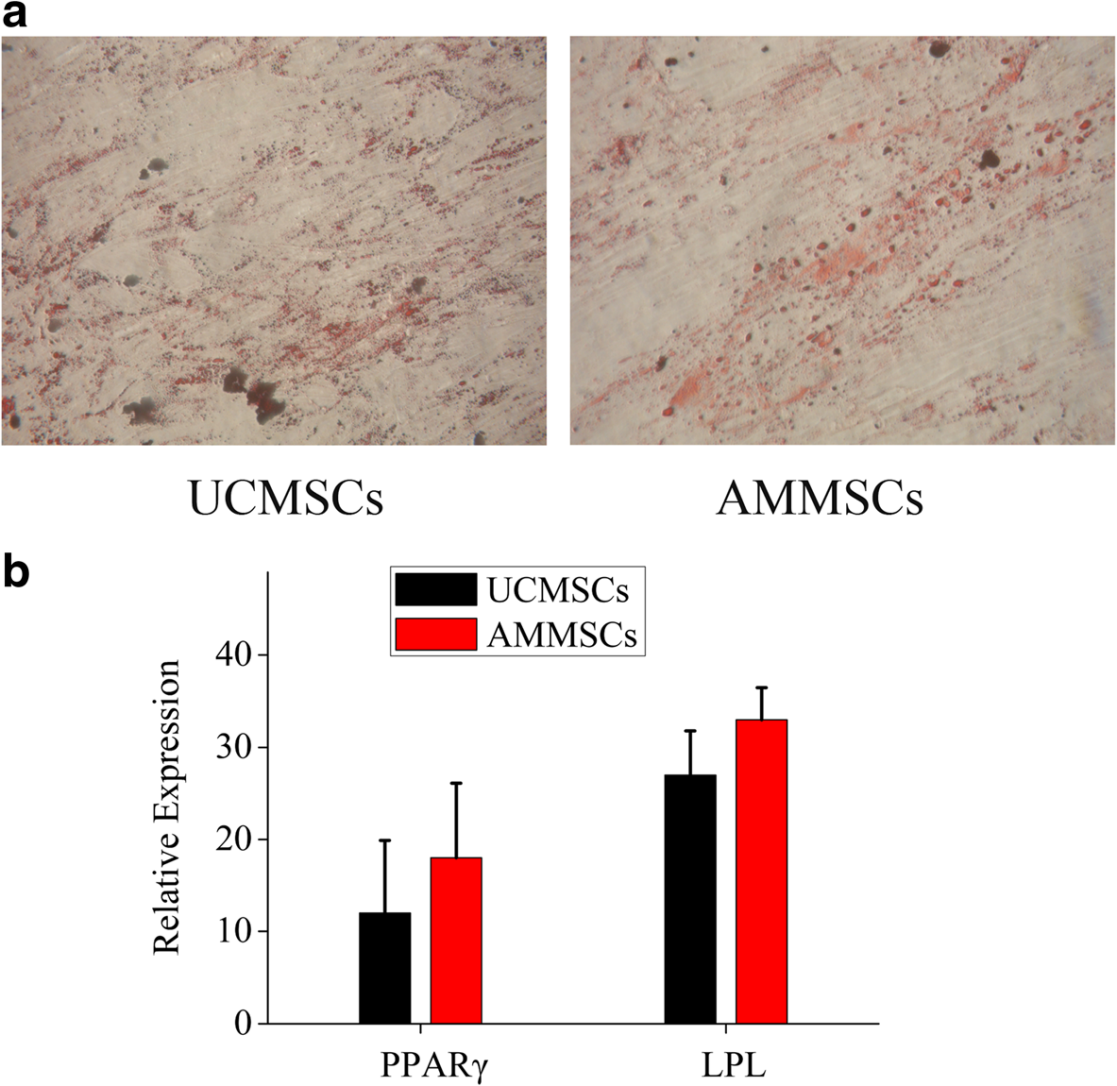
AMMSCs in PL showed similar adipogenic differentiation potential with UCMSCs. **a** The adipogenic differentiation of AMMSCs and UCMSCs was confirmed by Oil Red O staining of lipid vacuoles. Magnification × 100. **b** Comparative investigation of adipogenic differentiation capability of UCMSCs and AMMSCs was assessed by quantitative analysis of PPARg and LPL mRNA expression. Bars represented means ± SD; *n* = 5

The adipogenic differentiation of AMMSCs and UCMSCs was confirmed by oil red O staining of lipid vacuoles (Fig. 5a). The adipogenic AMMSCs exhibited statistically similar mRNA expression of PPARg and LPL with UCMSCs, indicating that AMMSCs in PL had similar adipogenic differentiation potential with UCMSCs (both *P* > 0.05, Fig. 5b).

### AMMSCs in PL showed superior immunosuppression effects with UCMSCs

**Fig. 6:**
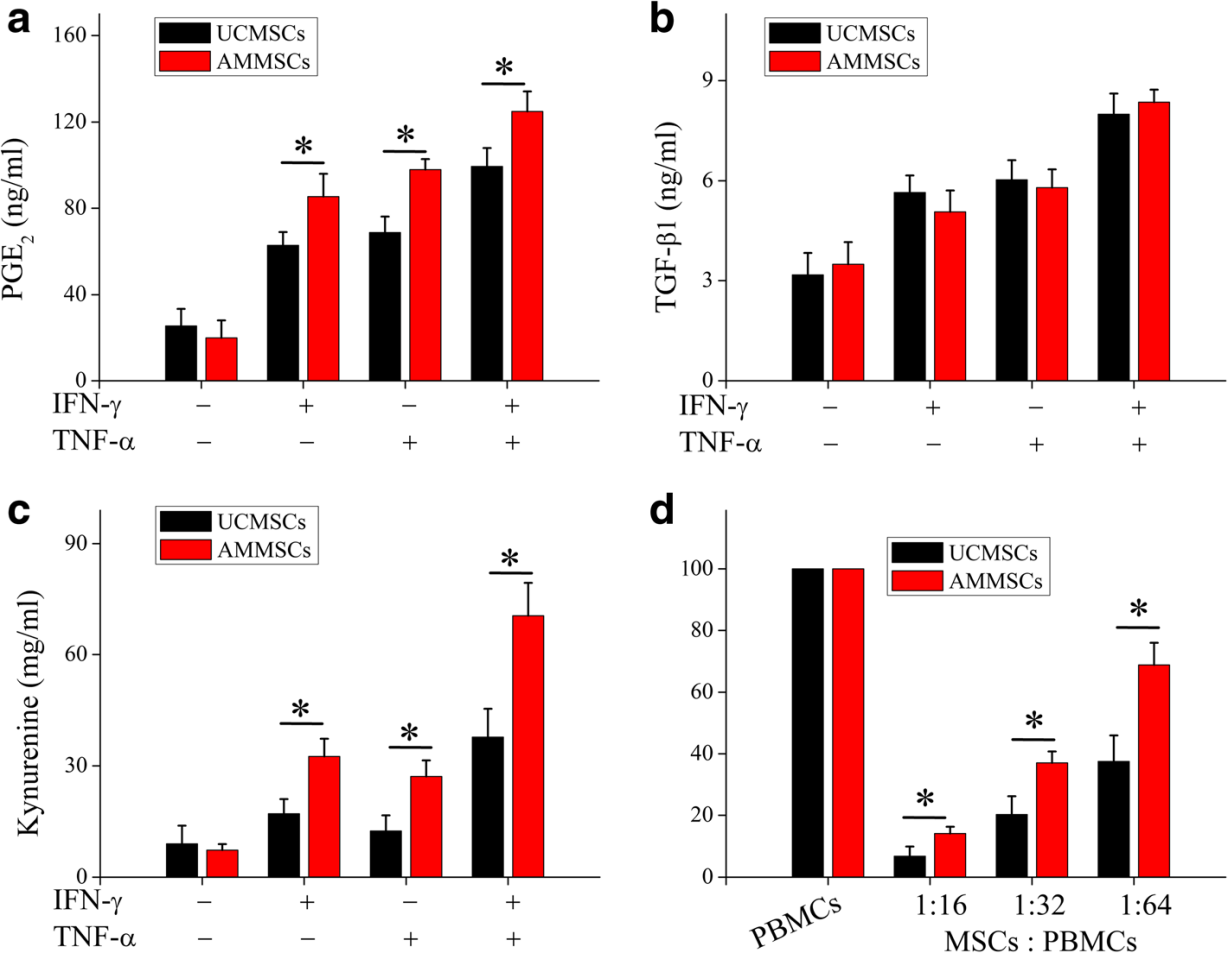
AMMSCs in PL showed superior immunosuppression effects with UCMSCs. When primed by IFN-γ and/or TNF-α induction, PGE_2_ (**a**) and TGF-β1 (**b**) expression were analyzed. IDO activity (**c**) was evaluated by kynurenine levels. (**d**) UCMSCs suppressed allogeneic lymphocyte proliferation. Bars represented means ± SD, *n* = 5; *P < 0.05

When no induction, both UCMSCs and AMMSCs exhibited a certain concentration of immunosuppression related mediators, and no significant differences in PGE_2_ and TGF-β1 expression (both *P* > 0.05, Fig. 6a and b) and IDO activity (*P* > 0.05, Fig. 6c) were observed between both types of cells. When primed by IFN-γ and (or) TNF-α induction, PGE_2_ and TGF-β1 expression levels and IDO activity were increased in UCMSCs and AMMSCs, and AMMSCs exhibited higher PGE_2_ expression (all *P* < 0.05, Fig. 6a) and IDO activity (all *P* < 0.05, Fig. 6c), but similar TGF-β1 expression (all *P* > 0.05, Fig. 6b) compared with UCMSCs. Moreover, AMMSCs showed a higher inhibitory effect on PBMCs proliferation than UCMSCs at different MSCs/PBMCs ratios (all *P* < 0.05, Fig. 6d). These results demonstrated that AMMSCs in PL had superior immunosuppression effects with UCMSCs.

## Discussion

Great success has been reported that PL is an increasingly alternative to FBS as a medium supplement for clinical grade expansion of MSCs for therapeutic applications [3, 20]. The growth factors including PDGF, TGF-β1, EGF and bFGF have been described as mitogens for MSCs [21], and bFGF and PDGF in PL are essential components for the growth-promoting effect of MSCs [22]. Our results show that PPP contains a certain concentration of these growth factors, but could not isolate and expand UCMSCs or AMMSCs in our previous studies (data not shown), demonstrating that low levels of these growth factors are not sufficient for MSCs proliferation. Freeze-thaw rupture of platelets had significantly elevated levels of growth factors in PL, which allowed expansion of UCMSCs and AMMSCs. These results demonstrate that the high concentrations of growth factors in PL are necessary for the proliferation of MSCs. Moreover, these growth factors in synergy with each other promote the proliferation of MSCs. Similar results were already described by others, although the concentrations reported by the various groups differ substantially [23–25]. It is known there is a homogenization of MSCs along time in culture, especially the method without cell sorting. In this study, both UCMSCs and AMMSCs exhibit heterogeneous morphology with various shapes in the early passages, showing low purity. The possibility of some resting mature cells layer in the initial adherent cells and the successive passages leads to a large amount of pure MSCs. However, due to genetic stability in culture, it is recommended by regulatory authorities not to use MSCs from late passages [26]. Moreover, we also found that aging cells appear after passage 5 in a previous study. Therefore, we suggest that MSC at passage 5 is used for evaluation of their characteristics. Similar plot was reported in previous studies [3, 19, 27]. In addition, MSCs-based therapies need viable and ample numbers of MSCs, a patient needs approximately 5 × 10^7^~5 × 10^8^ MSCs per transplantation, and usually multiple transplantation. In this study, a sufficient quantity of UCMSCs or AMMSCs can be collected in PL-supplemented cultures in 5 passages at a seeding density of 2000 cells/cm^2^. Based on a clinical point of view, passage 5 is more suitable than the earlier passage.

The basic biological characteristics including morphology, phenotype and differentiation capacity have been proposed as minimal criteria for defining MSCs by the International Society for Cellular Therapy (ISCT) [28]. Our results show that both UCMSCs and AMMSCs in PL fulfill all criteria, and no significant differences in these characteristics between both types of cells. It has been described that both UC and AM are derived from the foetus, this may be the reason why AMMSCs show similar biological characteristics with UCMSCs. Similar performance has been demonstrated between the two types of cells in FBS-supplemented medium [14, 15, 29, 30], but significant differences in morphology, phenotype and trilineage differentiation have been also revealed in FBS in some conflict studies [16, 17]. These apparent differences may be due to different isolation methods of MSCs. It has become widely assumed that besides source and culture condition, isolation methods of is critical in determining the characteristics and therapeutic potential of MSCs [13, 31, 32]. The self-renewal capacity is also one accepted characteristic of adherent MSCs in culture. Our results demonstrate that AMMSCs in PL showed similar self-renewal capacity with UCMSCs, consistent with the FBS-based comparative study [17]. Moreover, the cell population doubling time of the UCMSCs and AMMSCs in cultures supplemented with PL is in the range of 25-45 h based on our calculation, no significantly differences between two types of cells (data not shown). Compared the results with others available in literature, UCMSCs and AMMSCs in PL showed less cell population doubling time than those in FBS (the range of 45-60 h [16, 17]), showing that higher proliferative capacity in PL, respectively. However, a recent study has reported that AMMSCs in FBS show significantly lower proliferation than UCMSCs [14, 16]. This different proliferation rates are related to different isolation methods or culture condition. In addition, A possible explanation might be that AM includes two different stem cell populations showing distinct biological characteristics [33].

Increasing evidence in animal models of immune diseases supports the notion that the mechanism of functional benefit of MSCs is predominantly dependent on immunosuppression activity [34]. Thus, the ISCT proposes immune functional assays as potency release criterion for MSCs in 2013 and 2016 [35, 36]. When in a pro-inflammatory environment, MSCs could induce anti-inflammatory polarization. Moreover, the activation is necessary for the manifestation of immunosuppressive properties of MSCs. The ISCT suggests that in vitro experimental model with IFN-γ and/or TNF-α priming is used as a standard assay for the assessment of immune function of MSCs [35]. In-vitro inflammatory priming of MSCs could, to a certain extent, mimic what happens in vivo when MSCs are transplanted into patients with immune disorders [37]. It has become widely accepted that the addition of IFN-γ with or without TNF-α for 12–48 h is adequate to activate MSCs for the analysis of immunosuppression effect, which is mainly through the secretion of immunosuppression related mediators such as TGF-β1, PGE_2_ and IDO [34, 37]. Interestingly, our result shows that when no induction, similar levels of PGE_2_ and TGF-β1 expression and IDO activity are observed between UCMSCs and AMMSCs in PL, which is in conflict with the FBS-based comparative analysis described recently [17, 29]. One study has showed that AMMSCs are significantly higher production of TGF-β compared to UCMSCs [25], but another study shows opposite result [23]. These apparent differences may be due to different isolation methods and (or) culture condition of MSCs. In short, both UCMSCs and AMMSCs are to some extent naturally immunosuppressive capabilities without the need for priming, but independent of the culture conditions. Similar capabilities of UCMSCs in FBS-supplemented media have been also demonstrated by assessing anti-inflammatory cytokines in a recent study [38]. We also demonstrate that the priming of UCMSC and AMMSCs leads to an increase in the expression levels of immunosuppression related mediators, showing that the pre-activation could improve the immunosuppression capacity of two types of cells. When primed by IFN-γ and (or) TNF-α induction, AMMSCs exhibit higher PGE_2_ expression and IDO activity than UCMSCs, showing that AMMSCs are more potent immunosuppressors than UCMSCs. This result is in agreement with FBS-based comparison in previous reports [39, 40], but in contrast with data from other laboratory [41]. However, similar TGF-β1 expression is observed between both types of cells, this may be related to the high level of TGF-β1 in PL (Fig. 1b). Importantly, our result further demonstrates that AMMSCs are more potent immunosuppressors by inhibiting PBMCs proliferation effect, which contradicts the FBS-based comparative studies [14]. A possible explanation might be that AMMSCs in FBS are the most heterogeneous population due to low proliferative rate [16]. The high proliferative rate of AMMSCs in PL could lead to a reduction in proportion of some resting epithelial cells within the MSCs layer, and obtain a large amount of pure AMMSCs after the culture of successive passages.

## Conclusions

In summary, we conduct a direct head-to-head comparison with regard to biological characteristics and immunosuppression effects of AMMSCs and UCMSCs expanded in PL. Our results indicate that AMMSCs in PL have similar morphology, immunophenotype, self-renewal capacity, and trilineage differentiation potential, but superior immunosuppression effects in comparison with UCMSCs. Therefore, we hypothesize that AMMSCs might be more suitable than UCMSCs for treatment of immune diseases. Furthermore, in vivo functional studies are needed to confirm the prediction.

## Additional file

Additional file 1: Primer sequences for Real Time PCR analysis and their respective product sizes. (DOCX 13 kb)

## Abbreviations

AM: Amniotic membrane
AMMSCs: Amniotic membrane derived messechymal stem cells
bFGF: Basic fibroblast growth factor
CFU-F: Colony-forming unit fibroblast
CPD: Cumulative population doublings
EGF: Epithelial growth factor
ELISA: Enzyme linked immunosorbent assay
FBS: Fetal bovine serum
IDO: Indoleamine 2, 3-dioxygenase
IFN-γ: Interferon-gamma
IGF-1: Insulin-like growth factor-1
IMDM: Iscove’s modified dulbecco’s medium
ISCT: The International Society for Cellular Therapy
MSCs: Mesenchymal stem cells
PBMCs: Peripheral blood mononuclear cells
PBS: Phosphate buffered saline
PCR: Polymerase chain reaction
PD: Population doubling
PDGF: Platelet-derived growth factor
PGE_2_: Prostaglandin E_2_
PL: Platelet lysate
PPP: Platelet-poor plasma
PRP: Platelet-rich plasma
SD: Standard deviation
TGF-β1: Transforming growth factor-beta 1
TNF-α: Tumor necrosis factor-alpha
UC: Umbilical cord
UCMSCs: Umbilical cord derived messechymal stem cells
VEGF: Vascular endothelial growth factor
