# Supplementary material for: Comparative evaluation of mesenchymal stromal cells from umbilical cord and amniotic membrane in xeno-free conditions
Source: BMC Cell Biol. 2018 Dec 13;19:27. doi: 10.1186/s12860-018-0178-8 (PMC6293527; doi:10.1186/s12860-018-0178-8)
Supplement: Supplementary file 1 — Primer sequences for Real Time PCR analysis and their respective product sizes. (DOCX 13 kb) [file 12860_2018_178_MOESM1_ESM.docx]

**Additional file 1:** Primer sequences for Real Time PCR analysis and their respective product sizes.

| **Gene** | **Primer sequence (5' to 3')** | **Productsize (bp)** |
| --- | --- | --- |
| RUNX-2 | GGAGTGGACGAGGCAAGAGTTT | 133 |
|  | AGCTTCTGTCTGTGCCTTCTGG |  |
| Alkaline phosphatase | GGGAACGAGGTCACCTCCAT | 67 |
|  | TGGTCACAATGCCCACAGAT |  |
| SOX-9 | GTACCCGCACTTGCACAAC | 139 |
|  | GTAATCCGGGTGGTCCTTCT |  |
| Collagen II | GGCAATAGCAGGTTCACGTACA | 79 |
|  | CGATAACAGTCTTGCCCCACTT |  |
| PPARγ | GGCTTCATGACAAGGGAGTTTC | 74 |
|  | AACTCAAACTTGGGCTCCATAAAG |  |
| LPL | GAGGTACTTTTCAGCCAGGATGTAAC | 82 |
|  | AGCTGGTCCACATCTCCAAGTC |  |
| β-actin | TGACGTGGACATCCGCAAAG | 205 |
|  | CTGGAAGGTGGACAGCGAGG |  |
